# Gluteal Hydatid Cyst: Report of a Case from Iran

**Published:** 2017

**Authors:** BEHZAD NEMATI HONA, Farbod EMAMI YEGANEH

**Affiliations:** Dept. of General Surgery, Imam Hossein Hospital, Shahid Beheshti University of Medical Sciences, Tehran, Iran

**Keywords:** Gluteal, Hydatid cyst, Iran

## Abstract

Hydatid cyst involves both hard and soft tissues even without the evidence of the disease in liver or lungs; however, this manifestation is very rare, particularly in musculoskeletal regions). The current report describes a case with primary diagnose of cystic gluteal swelling leading to diagnose of hydatid cyst after surgical exploration in an 80-yr-old woman the Surgical Outpatient Department, Imam Hossein Hospital, Shahid Beheshti University of Medical Sciences, Tehran, Iran. During surgery, the cavity was washed by silver nitrate and the cyst content was appropriately evacuated. The patient had completed a short course of albendazole postoperatively. Early postoperative complications were not appeared. The patient was followed for 6 months with no evidence of recurrence or complications.

## Introduction

Hydatid cyst is a fairly common parasitic disease caused by the infestation of a worm named Echinococcus to different body organs (1). Most cases with hydatid cyst remain latent even in older adults; however, it may be symptomatic by parasite overload, increasing the size of cysts, or appearing the cyst in unusual parts of the body (2). Thus, echinococcosis can theoretically involve any body organ mostly in liver (63%), lungs (25%), and muscles (5%); however, it can also occur in skeletal system, kidneys, brain, spleen, and even eyes (3). Thus, involvement in any organ leads to the appearance of specific manifestations of that organ. Overall, soft tissues especially peripheral muscles are rare sites of hydatid cyst counting for less than 1% of total cases (4).

The current report describes a case of cystic gluteal swelling turning out to be hydatid cyst on surgical exploration, which is a very rare occurrence.

## Case Presentation

An 80-year-old woman presented to the surgical outpatient department with a slowly growing painful swelling in the left gluteal region for the past 2 months. The patients had the history of repeated muscular injections. She experienced surgical discharge and drainage of hydatid cyst of the liver 10 years ago. The patients had also the history of hypertension, diabetes mellitus, and myocardial infarction. Regarding vital sign on admission, the patient was stable with the blood pressure of 150/90 mmHg, pulse rate of 80/min and temperature of 37 °C. Informed consent was taken from the patient.

**Fig. 1:**
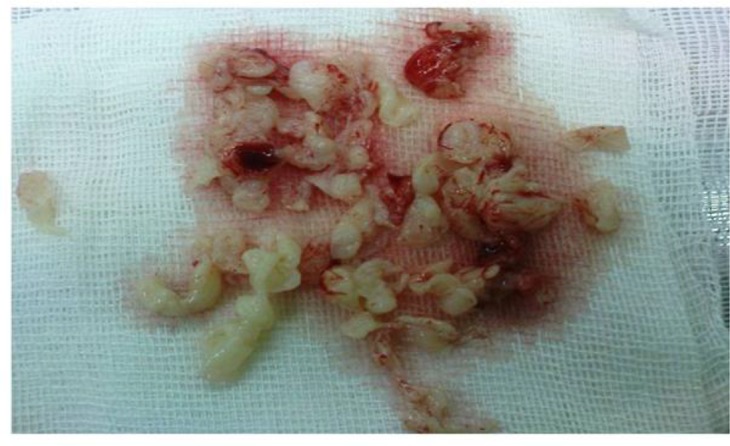
Fragmented tissues evacuated from the cyst (Original)

**Fig. 2:**
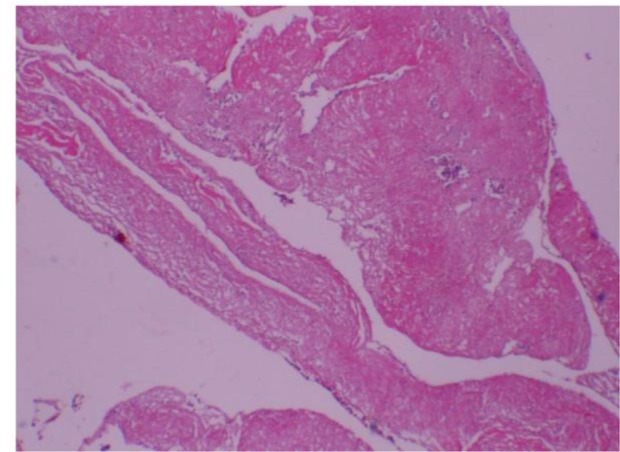
Histological View of hydatid cyst (Original)

In physical examination, a midline laparotomy scar from previous surgery was observed. In addition, it was revealed a tender, fluctuated cystic swelling fixed to left gluteal muscle with evidence of local inflammation. This was followed by surgical drainage of the mass. The patient was operated under a spinal anesthesia. A primary differential diagnosis of gluteal abscess was considered, but after surgical incision, a turbid liquid containing yellow fragmented tissues leaked from the cyst leads to diagnosis of hydatid cyst (Fig. 1, 2).

**Fig. 3:**
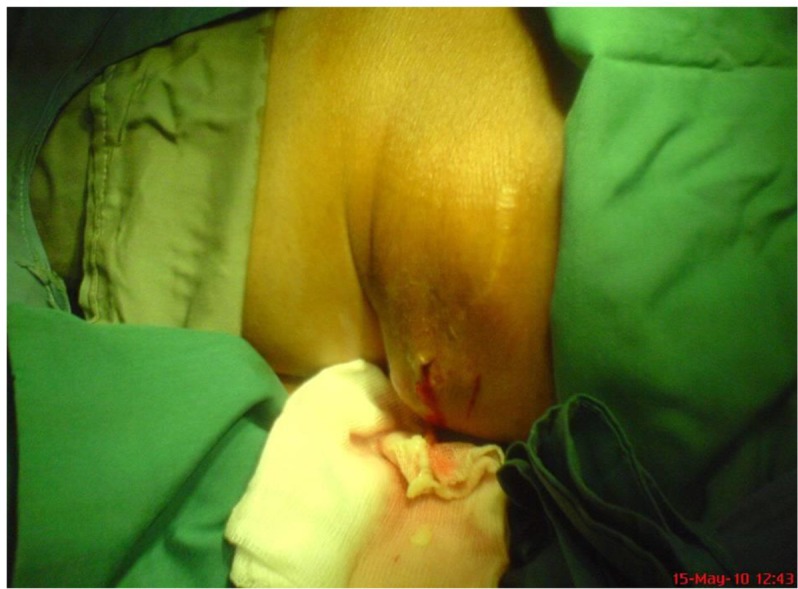
Washing the cavity by silver nitrate and drainage of the liquid from that (Original)

Then, the cavity was washed by silver nitrate and the liquid was appropriately drained (Fig. 3). The patient had completed a short course of albendazole postoperatively. Early postoperative complications were not appeared. The patient was followed for 6 months with no evidence of recurrence or complications.

## Discussion

Musculoskeletal hydatid cyst is a rare entity, which can be considered as a differential diagnosis for any painful mass in gluteal muscle in patients presented with previous history of hydatid cyst (3, 4).

The diagnosis of hydatid cyst is based on discovering a hydatid cyst in suspected tissue; however diagnosis can be confirmed by some supplemental diagnostic methods such as serological tests, fine needle aspiration (FNA), and imaging modalities (5,6). In large symptomatic cysts (especially greater than 5cm), surgical interventions may be required as cyst drainage and washing the affected cavity ( 7Cyst rupture may result in anaphylactic shock or releasing scoleces that implant elsewhere and produce secondary cysts (8). As medical approach, Albendazole is a useful option in treating lesion, with high scolicidal effect (9).

In total, hydatidosis affecting the muscles without the evidences of liver or lung involvements is a rare diagnosis. A primary case of hydatid cyst was recently reported (10) in a woman with hydatid cyst located in gluteal muscle that was completely excited by surgery and albendazole was prescribed preoperatively and postoperatively to reduce risk for local recurrence. Gürbüz et al. (11) also recently reported another case of gluteal hydatid cyst that was successful managed surgical without any postoperative complication. Sreeramulu et al. (12) also reported a case of 34-year-old male patient with a cystic gluteal swelling turning out to be hydatid cyst on surgical exploration that was completely removed by surgical management in combination with a pharmacology complementary treatment with antihelementhics. Haque et al. (13) described a case of a 24-year-old male patient with a cystic gluteal-swelling turning out to be hydatid cyst that was finally diagnosed using sonography, computerized scanning, and treated with surgical drainage combined with postoperative antihelmenthics.

In the current study, the diagnosis was directly based on surgical excision and the treatment was planned on surgical drainage, washing the cavity with silver nitrate, and short-course administration of Albendazole led to proper long-term outcome.
